# The MITF-SOX10 regulated long non-coding RNA DIRC3 is a melanoma tumour suppressor

**DOI:** 10.1371/journal.pgen.1008501

**Published:** 2019-12-27

**Authors:** Elizabeth A. Coe, Jennifer Y. Tan, Michael Shapiro, Pakavarin Louphrasitthiphol, Andrew R. Bassett, Ana C. Marques, Colin R. Goding, Keith W. Vance

**Affiliations:** 1 Department of Biology and Biochemistry, University of Bath, Bath, United Kingdom; 2 Department of Computational Biology, University of Lausanne, Lausanne, Switzerland; 3 Ludwig Institute for Cancer Research, University of Oxford, Oxford, United Kingdom; 4 Wellcome Sanger Institute, Wellcome Genome Campus, Hinxton, United Kingdom; Brigham and Women’s Hospital, UNITED STATES

## Abstract

The MITF and SOX10 transcription factors regulate the expression of genes important for melanoma proliferation, invasion and metastasis. Despite growing evidence of the contribution of long noncoding RNAs (lncRNAs) in cancer, including melanoma, their functions within MITF-SOX10 transcriptional programmes remain poorly investigated. Here we identify 245 candidate melanoma associated lncRNAs whose loci are co-occupied by MITF-SOX10 and that are enriched at active enhancer-like regions. Our work suggests that one of these, *Disrupted In Renal Carcinoma 3* (*DIRC3*), may be a clinically important MITF-SOX10 regulated tumour suppressor. *DIRC3* depletion in human melanoma cells leads to increased anchorage-independent growth, a hallmark of malignant transformation, whilst melanoma patients classified by low *DIRC3* expression have decreased survival. *DIRC3* is a nuclear lncRNA that activates expression of its neighbouring *IGFBP5* tumour suppressor through modulating chromatin structure and suppressing SOX10 binding to putative regulatory elements within the *DIRC3* locus. In turn, *DIRC3* dependent regulation of *IGFBP5* impacts the expression of genes involved in cancer associated processes and is needed for *DIRC3* control of anchorage-independent growth. Our work indicates that lncRNA components of MITF-SOX10 networks are an important new class of melanoma regulators and candidate therapeutic targets that can act not only as downstream mediators of MITF-SOX10 function but as feedback regulators of MITF-SOX10 activity.

## Author summary

The human genome expresses thousands of long non-coding RNAs (lncRNAs), in addition to protein coding genes. Although the majority of these molecules are of unknown function, a subset of lncRNAs have been proposed to represent a new class of cancer causing genes. While lncRNAs in melanoma have great potential for the development of new treatments, it remains unclear how they integrate into the well-defined gene expression networks controlling the formation and progression of this disease. Here we have identified a new set of 245 candidate melanoma-associated lncRNAs that are targeted by the MITF and SOX10 melanoma transcription factors. We show that one of these, *DIRC3*, may be a clinically important MITF-SOX10 regulated melanoma tumour suppressor that acts to block the spread of the disease and that melanoma patients with low *DIRC3* expression have decreased survival. We also discovered that *DIRC3* functions as a new transcriptional regulator to activate its neighbouring *IGFBP5* gene and control genome-wide expression programmes involved in cancer. Our results suggest that lncRNA components of MITF-SOX10 networks represent an important new class of melanoma regulators and predict that up-regulation of *DIRC3* expression may represent an exciting new therapeutic strategy for melanoma.

## Introduction

Growing evidence supports the biological relevance of gene expression regulation by long non-coding RNAs (lncRNAs) in health and disease. Whilst the molecular functions of most lncRNAs remain poorly understood, investigation of the mode of action of a small number of these transcripts has revealed that they contribute to multiple aspects of gene expression output [1, 2]. Specifically, a subset of nuclear lncRNAs control the expression of adjacent genes by acting locally, close to their sites of synthesis. Such functions can be mediated by transcript-dependent interactions to facilitate the recruitment of transcription/chromatin regulatory proteins to neighbouring genomic sites and/or to modulate ribonucleoprotein complex assembly [3, 4]. Alternatively, some nuclear lncRNAs have transcript-independent functions. These can be mediated by the process of RNA polymerase II transcription or splicing, or by the action of DNA-dependent regulatory elements within their loci [5, 6]. In addition, a small number of lncRNA transcripts act in *trans* by moving away from their sites of synthesis to bind and directly regulate multiple target genes on different chromosomes [7–9].

Given their critical regulatory roles, not surprisingly a subset of lncRNAs have been recurrently implicated in cancer. For example, 14.6% of lncRNA loci are found within focal somatic copy number alterations, containing no cancer-associated protein coding genes, in cancer cell genomes [10]. LncRNA expression is also dysregulated in most cancers, including melanoma, and lncRNA expression signatures can be used to distinguish different cancers from their normal tissue types [10, 11]. LncRNAs therefore have potential as cancer biomarkers. A small number of lncRNAs can also function as oncogenes or tumour suppressors and act as key components of gene regulatory networks controlling carcinogenesis. Such lncRNAs may represent novel therapeutic targets for the development of cancer treatments. For example, activation of the p53 tumour suppressor induces the expression of hundreds of lncRNAs. One of these, *lincRNA-p21* (*TP53COR1*), is needed for p53 dependent repression of anti-apoptotic genes whilst another, *LINC-PINT*, mediates p53-dependent cell cycle arrest [12, 13]. Similarly, the MYC oncoprotein binds the promoters of 616 lncRNAs in human B cells, where it directly regulates the expression of some of these [14]. Two MYC regulated lncRNAs, *MYCLo-1* and *MYCLo-2*, have also been shown to repress genes that promote colorectal cancer cell proliferation in a MYC-dependent manner [15]. These examples illustrate the importance of lncRNA gene regulatory functions downstream of tumour suppressor and oncogenic transcription factors.

Over recent years melanoma has emerged as a leading model for cancer progression, especially with regard to understanding how the microenvironment plays a key role in generating the phenotypic heterogeneity that drives disease progression. It is now apparent that lncRNAs can exert important roles in the biology of melanoma. For example, oncogenic BRAF^V600E^ signalling regulates the expression of 109 putative lncRNAs, one of which *BANCR*, functions as a melanoma oncogene to activate gene expression programmes controlling cell migration [16]. Another lncRNA, *MIR31HG*, induces *INK4A*-dependent senescence in response to BRAF^V600E^ expression [17], whereas the lncRNA *SAMMSON* is needed for melanoma proliferation and survival. Targeted inhibition of *SAMMSON* disrupts mitochondrial function and increases the response to MAPK-inhibitor drugs in patient-derived xenograft models of melanoma [18]. However, while melanoma associated lncRNAs have great potential for the development of new melanoma treatments, how lncRNAs integrate into the well-defined gene expression networks that underpin different phenotypic states in melanoma is less clear.

The microphthalmia-associated transcription factor MITF plays a critical role in melanocyte development and in melanoma. MITF activates the expression of protein coding genes involved in differentiation and proliferation, DNA replication and repair, mitosis, oxidative phosphorylation and mitochondrial metabolism, and represses the transcription of genes involved in melanoma cell invasion and motility [19–23]. *MITF* amplification is frequent in melanoma and particularly common in metastatic forms of the disease where it associates with poor survival [24]. *MITF* expression is controlled in part through the action of SOX10 [25–28], and together MITF-SOX10 co-occupy several thousand binding sites on chromatin to control key gene regulatory networks governing melanocyte development and melanoma [29]. Recent work has also reported that MITF-SOX10 drive the proliferative cell state in melanoma and influence the response to MAPKinase inhibiting therapeutics [30, 31]. Although the SOX10-regulated lncRNA *SAMMSON* is co-amplified with *MITF* in melanoma [18], our understanding of how MITF-SOX10 drive melanocyte and melanoma biology, and how much of their activity is mediated by lncRNAs is largely unexplored.

Here we identify 245 candidate melanoma associated lncRNAs that are targeted by MITF-SOX10. We show that one of these, *Disrupted In Renal Carcinoma 3* (*DIRC3)*, may be a clinically important MITF-SOX10 repressed melanoma tumour suppressor gene that inhibits anchorage-independent growth. Our results reveal that *DIRC3* activates expression of the neighbouring *Insulin Like Growth Factor Binding Protein 5 (IGFBP5)* tumour suppressor gene to control gene expression programmes involved in cancer. Our work highlights that MITF-SOX10 bound lncRNAs, as exemplified by *DIRC3*, can function as important downstream components and feedback regulators of MITF-SOX10 expression networks in the control melanoma growth and progression.

## Results

### Identification of new melanoma-associated lncRNAs targeted by MITF-SOX10

MITF-SOX10-regulated transcriptional programmes play critical roles in the control of melanoma proliferation, invasion and metastasis. Although protein coding gene networks involved in mediating the downstream MITF-SOX10 transcriptional response have been well-defined, the identity and putative functions for MITF-SOX10 regulated lncRNAs in melanoma remain unknown.

**Fig 1:**
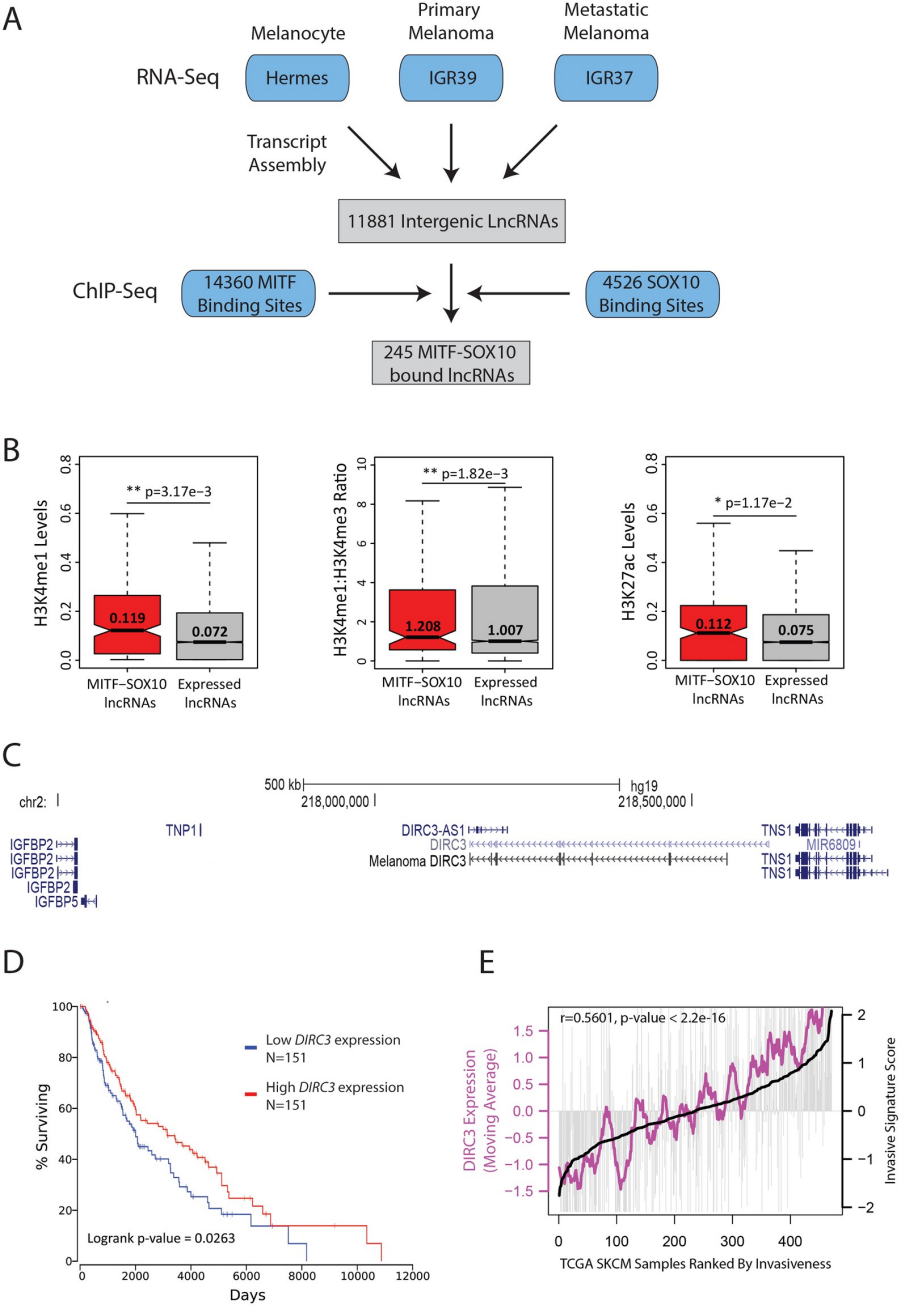
LncRNAs are components of MITF-SOX10 networks in melanoma. (A) Workflow diagram describing the experimental and computational methods used to identify a set of melanocyte and/or melanoma expressed intergenic lncRNAs whose genomic loci are bound by the MITF and SOX10. (B) Distribution of the number of normalised H3K4me1 (left panel), H3K27ac (right panel), and ratio of H3K4me1 to H3K4me3 (middle panel) sequencing reads mapped to MITF-SOX10 bound lncRNAs (red) and all expressed lncRNA (grey) loci in the sh-PTEN HMEL tumorigenic melanoma cell line [32]. Differences between groups were tested using a two-tailed Mann-Whitney *U* test, and *p* values are indicated. (C) Schematic illustration of the human *DIRC3* locus and neighbouring protein coding genes (GRCh37/hg19). Rapid amplification of cDNA ends (RACE) and RT-PCR experiments defined *DIRC3* as a 3,384 nucleotide multi-exonic transcript in human melanoma cells. (D) TCGA survival data for Skin Cutaneous Melanoma (SKCM) was linked to *DIRC3* expression using OncoLnc [33]. Patients were sorted based on *DIRC3* expression and percent survival compared between *DIRC3* high (top third) and *DIRC3* low (bottom third) groups. Cox regression analysis shows that low *DIRC3* expression correlates with statistically significant decreased survival in melanoma patients (logrank p-value = 0.0263). (E) *DIRC3* levels correlate with an invasiveness gene expression signature [31] in melanoma. The 471 TCGA human melanoma RNA-seq samples were ranked by increasing invasion signature score. Vertical grey lines indicate *DIRC3* expression in each melanoma sample. Moving averages are plotted in bold.

To identify candidate lncRNA regulators of melanoma that are part of the MITF-SOX10 network, we used RNA-sequencing (RNA-seq) of Hermes immortalised human melanocytes and two human BRAF^V600E^ mutated melanoma cell lines isolated from the same patient: IGR39 which are MITF-low, de-differentiated and invasive and IGR37 which are MITF-high and non-invasive. This identified a comprehensive set of 11881 intergenic lncRNA transcripts expressed in at least one of these cell types (Fig 1A, S1 Table). Integration of genome wide maps of HA-tagged-MITF and SOX10 binding in human melanoma from [29] then defined a stringent set of 245 candidate melanoma-associated lncRNAs whose genomic loci are co-occupied by these two transcription factors (Fig 1A, S2 Table). Using chromatin state maps from tumorigenic melanocytes [32], we determined that compared to all other melanocyte and melanoma expressed lncRNAs, MITF-SOX10 bound lncRNA loci displayed increased levels of H3K27ac and H3K4me1 modified chromatin, as well as a corresponding higher ratio of the H3K4me1:me3 enhancer-associated chromatin signature, (p<0.05, two-tailed Mann Whitney U test, Fig 1B and S1 Fig). This suggests that MITF-SOX10 bound lncRNAs are enriched at active transcriptional enhancer-like regions and we predict that a subset of these lncRNAs are likely to play an important role in mediating the MITF-SOX10 transcriptional response in melanoma.

To illustrate proof of concept that some MITF-SOX10 bound lncRNAs may comprise an important new class of melanoma regulators we prioritised *DIRC3*, a multi-exonic 3,384 nucleotide transcript in human melanoma cells (Fig 1C), for further investigation. This was based on the following reasons: (i) The Cancer Genome Atlas (TCGA) melanoma patients expressing low levels of *DIRC3* show statistically significant decreased survival compared to those classified based on high *DIRC3* (Fig 1D). (ii) The human *DIRC3* locus spans approximately 450 kb genomic sequence between the *IGFBP5* and *TNS1* genes (Fig 1C). Overexpression of the *IGFBP5* mediator of *insulin-like growth factor receptor* (*IGF1R*) signalling inhibits the transformation of human melanoma cells in culture whilst *TNS1* expression is down-regulated in multiple cancers including melanoma [34, 35]. This gene territory therefore encodes important tumour suppressive functions. (iii) *DIRC3* levels positively correlate with an invasiveness gene expression signature [31] across the 471 TCGA melanoma RNA-seq samples (Fig 1E). (iv) Expression of a positionally equivalent *DIRC3* orthologue in mice further indicated that *DIRC3* is a functionally important lncRNA (S2 Fig). Together, these data provide strong initial evidence that *DIRC3* may act as a clinically important new lncRNA regulator of melanoma.

### *DIRC3* expression is directly repressed by MITF and SOX10

**Fig 2:**
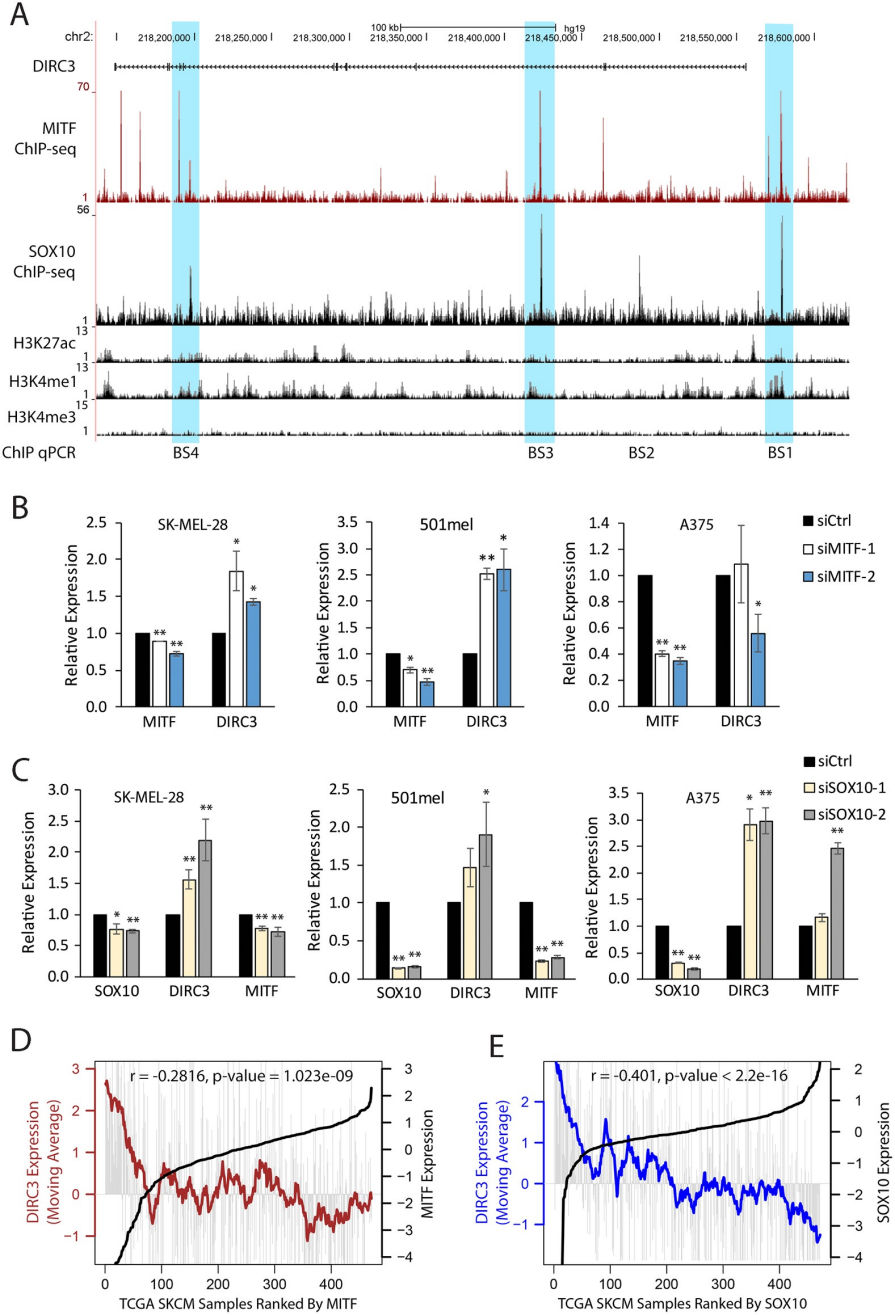
*DIRC3* is a direct MITF and SOX10 transcriptional target. (A) UCSC genome browser view showing that the *DIRC3* locus contains multiple ChIP-seq defined binding sites for MITF and SOX10 in 501mel cells [29]. MITF (B) and SOX10 (C) repress *DIRC3* in human melanoma cells. MITF and SOX10 were depleted in SK-MEL-28, 501mel and A375 cells using siRNA transfection. Expression changes were analysed using RT-qPCR. *POLII* was used as a reference gene. Results presented as mean +/- SEM., n≥3; one-tailed t-test * p<0.05, ** p<0.01. MITF and SOX10 protein levels are shown in S3 Fig. (D, E) *DIRC3* expression inversely correlates with *MITF* (D) and *SOX10* (E) in melanoma patients. *DIRC3* levels were analysed in 471 TCGA human melanoma samples ranked using increasing *MITF* or *SOX10*. Vertical grey lines indicate *DIRC3* expression in each melanoma sample. Moving averages are plotted in bold.

Analysis of chromatin modification [32] and MITF-SOX10 [29] binding profiles showed that the *DIRC3* locus contains three sites of MITF-SOX10 co-occupancy in melanoma: one upstream of the *DIRC3* TSS (BS1) and two within the *DIRC3* gene body (BS3-BS4, Fig 2A). These MITF-SOX10 bound sequences are located close to peaks of increased H3K27ac and H3K4me1 suggesting that they are involved in transcriptional control. To test if *DIRC3* is a MITF and SOX10 regulated target gene we first used multiple different siRNAs to deplete these two transcription factors in 501mel, SK-MEL-28 and A375 human melanoma cell lines, and measured changes in *DIRC3* expression using RT-qPCR. Changes in MITF and SOX10 protein levels are shown in S3 Fig. Reduction of MITF led to increased *DIRC3* expression in both 501mel and SK-MEL-28, but not in A375 cells (Fig 2B), indicating that *DIRC3* is transcriptionally repressed by MITF in at least a subset of melanoma cells, whilst SOX10 knockdown using two independent siRNAs increased *DIRC3* expression in all three melanoma cell lines tested (Fig 2C). *MITF* expression was significantly reduced in SK-MEL-28 and 501mel cells, but not in A375 cells, upon SOX10 depletion, suggesting that SOX10 mediated repression of *DIRC3* occurs, at least in part, independent from its regulation of *MITF*. Consistent with these findings, analysis of *DIRC3*, *MITF* and *SOX10* expression across the 471 TCGA melanoma RNA-seq samples showed that *DIRC3* levels negatively correlate with both *MITF* and SOX10 in melanoma patients, with *DIRC3* expression being especially high in those tumours with the lowest *MITF* (Fig 2D) or *SOX10* (Fig 2E) expression. Taken together, these data provide strong evidence that *DIRC3* is transcriptionally repressed by SOX10 and MITF in melanoma.

### *DIRC3* acts locally to activate expression of the *IGFBP5* tumour suppressor

**Fig 3:**
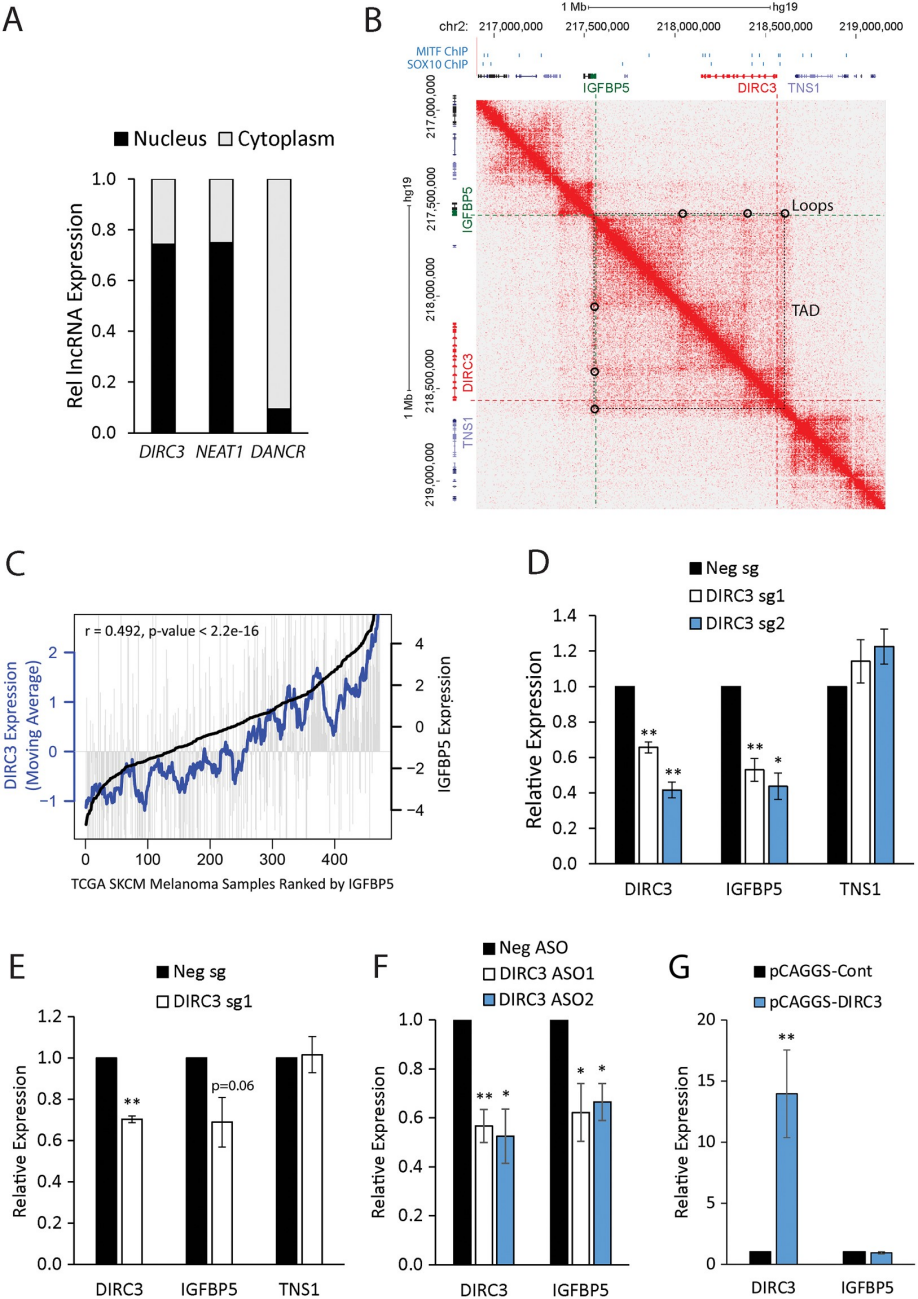
*DIRC3* acts locally to activate expression of the adjacent *IGFBP5* tumour suppressor gene. (A) *DIRC3* transcript is enriched in the nucleus in melanoma cells. SK-MEL-28 cells were biochemically separated into cytoplasmic and nuclear fractions. The relative levels of *DIRC3* and control *DANCR* (cytoplasm) and *NEAT1* (nuclear) transcripts in each fraction were determined by qRT-PCR. (B) *DIRC3* and *IGFBP5* are located within the same TAD. Heatmap displaying chromosomal interactions, measured using HiC, at regions surrounding *DIRC3* (red), *IGFBP5* (green) and TSN1 (purple), shown in gene browser view, in NHEK (chr2: 217,000,000–219,000,000). The dotted black square box on the heatmap represents a TAD. Chromosomal looping interactions are indicated by black circles. MITF and SOX10 binding sites are denoted as blue boxes. (C) *DIRC3* expression correlates with *IGFBP5* in melanoma patient samples. Analysis of *DIRC3* expression in TCGA human melanoma samples ranked by increasing *IGFBP5*. Vertical grey lines indicate *DIRC3* expression in each melanoma sample. Bold black indicates *IGFBP5* expression; the blue line is the moving average of *DIRC3* expression across 20 melanoma samples. (D-F) *DIRC3* specifically activates *IGFBP5* expression in a transcript dependent manner. *DIRC3* levels were depleted by dCas9-KRAB mediated CRISPRi (D), steric hindrance with dCas9 (E) or ASO LNA GapmeR mediated transcript degradation (F) in SK-MEL-28 cells. Expression of *DIRC3* and the indicated neighbouring genes were measured using RT-qPCR with results normalised to *POLII*. Expression changes are shown relative to a non-targeting control (set at 1). (G) Ectopic *DIRC3* fails to activate *IGFBP5*. SK-MEL-28 cells were transfected with pCAGGS-*DIRC3* or pCAGGS alone and cells harvested for expression analysis 3 days later. Results are presented relative to the pCAGGS control. For all RT-qPCR reactions, mean values +/- SEM are shown, n≥3. One-tailed student’s t-test p < 0.05 * p < 0.01 **

We next investigated whether *DIRC3* functions to regulate the expression of its adjacent protein-coding genes. Subcellular fractionation experiments first showed that *DIRC3* is a nuclear-enriched transcript in human melanoma cells suggesting that it may function as a novel transcriptional regulatory lncRNA (Fig 3A). Control *DANCR* and *NEAT1* lncRNAs were predominantly localised in the cytoplasm and nucleus respectively confirming the efficacy of the fractionation. We then mapped the chromatin structure of the *IGFBP5*-*DIRC3-TNS1* gene territory using Hi-C data from Normal Human Epidermal Keratinocytes (NHEK) [36]. This showed that *DIRC3* and *IGFBP5*, but not *TNS1*, are located within the same self-interacting topologically associated domain (TAD) and identified two DNA looping interactions between the *DIRC3* locus and the *IGFBP5* gene promoter (Fig 3B). This indicates that chromatin regulatory interactions bring *DIRC3* into close genomic proximity with *IGFBP5* in NHEK cells. Although, TAD structures can vary between normal tissues and cancers, a high proportion of TADs are invariable [37] and we found that *DIRC3* levels positively correlate with *IGFBP5* in TCGA melanoma RNA-seq samples (Fig 3C). These data therefore suggest a role for *DIRC3* in the local regulation of *IGFBP5* in melanoma.

To test whether *DIRC3* transcriptionally regulates *IGFBP5* or *TNS1*, *DIRC3* expression was reduced in SK-MEL-28 cells using CRISPR interference (CRISPRi) with two different sgRNAs (*DIRC3*sg1 and *DIRC3*sg2) to target the catalytically inactive dCas9-KRAB transcriptional repressor to the *DIRC3* promoter. RT-qPCR analysis of neighbouring gene expression showed that *DIRC3* depletion specifically down-regulated expression of the 3’ *IGFBP5* gene, when compared to a control non-targeting guide, whilst the levels of the 5’ *TNS1* gene did not change (Fig 3D). To validate the specificity of this regulation we then used the *DIRC3*sg1 RNA to precisely target dCas9 alone to the *DIRC3* TSS and block *DIRC3* transcription by steric hindrance of RNA polymerase recruitment. RT-qPCR confirmed that *DIRC3* down-regulation also reduced *IGFBP5* expression using this approach whilst *TNS1* levels did not change (Fig 3E). LncRNA gene regulatory functions can be mediated by the RNA molecule or by the process of RNA polymerase II transcription and/or splicing [2]. To test whether *DIRC3* function was transcript-dependent, we used locked nucleic acid (LNA)-modified anti-sense oligonucleotides (ASOs) to deplete *DIRC3* transcript levels and measured changes in *IGFBP5* in SK-MEL-28 cells. This showed that an approximately 50% reduction in the levels of *DIRC3* using two different ASOs resulted in a specific down-regulation of *IGFBP5* compared to a non-targeting control ASO (Fig 3F). Furthermore, nuclear lncRNAs can act either in *cis* or *trans* to regulate local gene expression [1, 2, 6, 38]. To investigate *DIRC3* mechanism of action, we expressed *DIRC3* from a transfected plasmid and discovered that exogenous *DIRC3* transcript fails to activate *IGFBP5* (Fig 3G). This is consistent with a model in which *DIRC3* acts *in cis* at its site of synthesis to control neighbouring gene expression.

Together, these data indicate that *DIRC3* is a nuclear localised transcriptional regulator that acts in *cis* in a transcript-dependent manner to activate expression of the adjacent *IGFBP5* tumour suppressor gene in melanoma.

### *DIRC3* regulates *IGFBP5*-dependent gene expression programmes involved in cancer

**Fig 4:**
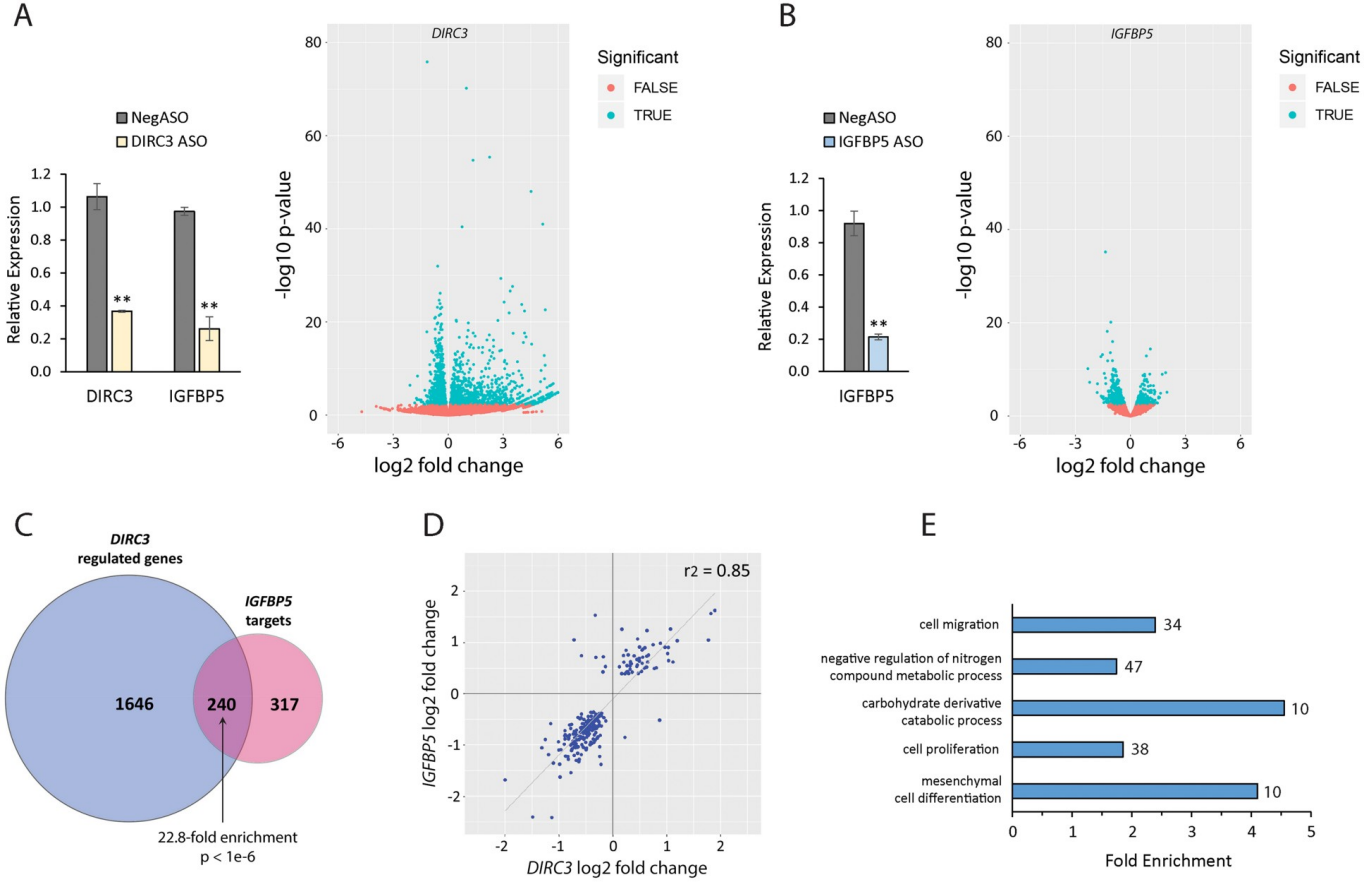
*DIRC3* regulates *IGFBP5*-dependent gene expression programmes involved in cancer. (A) *DIRC3* depletion induces statistically significant changes (FRD 5%) in the expression of 1886 genes in SK-MEL-28 cells using RNA-seq. (B) RNA-seq of *IGFBP5* knockdown SK-MEL-28 cells identifies 557 *IGFBP5* target genes (FDR 5%). (C) Intersection of *DIRC3*- and *IGFBP5*-regulated genes detects 240 common targets. (D) The expression levels of all *DIRC3*-*IGFBP5* shared target genes change in the same direction following either *DIRC3* or *IGFBP5* depletion. (E) Gene Ontology enrichment analysis of *DIRC3*- and *IGFBP5* shared target genes was performed using GOstats and FDR correction was applied. Representative significantly enriched categories are shown and the number of genes found in each category are indicated.

We next performed RNA-seq of *DIRC3* knockdown SK-MEL-28 cells to define the genome-wide impact of *DIRC3* expression in melanoma. ASO-mediated depletion of *DIRC3* expression by approximately 65% resulted in significant changes in the expression of 1886 genes (at a 5% false discovery rate (FDR)) compared to a non-targeting control (Fig 4A and S3 Table). 1015 (54%) of these genes were up-regulated and 871 (46%) were down-regulated upon *DIRC3* loss of function. To determine the extent by which *DIRC3* transcriptional response is mediated through *IGFBP5* regulation, we also performed RNA-seq of SK-MEL-28 cells in which *IGFBP5* expression was reduced by approximately 80%. This identified 557 differentially expressed genes (5% FDR) compared to a negative control (Fig 4B and S4 Table). We then analysed the intersection of *DIRC3* and *IGFBP5* regulated genes and identified 240 common targets (Fig 4C and S5 Table). This overlap represents a significant 22.8-fold enrichment (p < 1e-6) over the expected number based on random sampling of all expressed genes. Moreover, the expression levels of the majority of shared target genes change in the same direction following either *DIRC3* or *IGFBP5* depletion, providing further confirmation that *DIRC3* up-regulates *IGFBP5* (Fig 4D). Gene ontology (GO) enrichment analysis revealed that *DIRC3*-*IGFBP5* shared targets are significantly enriched for regulators of cell migration, proliferation, differentiation and metabolism (Fig 4E and S6 Table). *DIRC3* therefore possesses *IGFBP5*–dependent gene regulatory functions in melanoma that act to control cancer associated biological processes.

### *DIRC3* acts through *IGFBP5* to block anchorage-independent growth in melanoma

**Fig 5:**
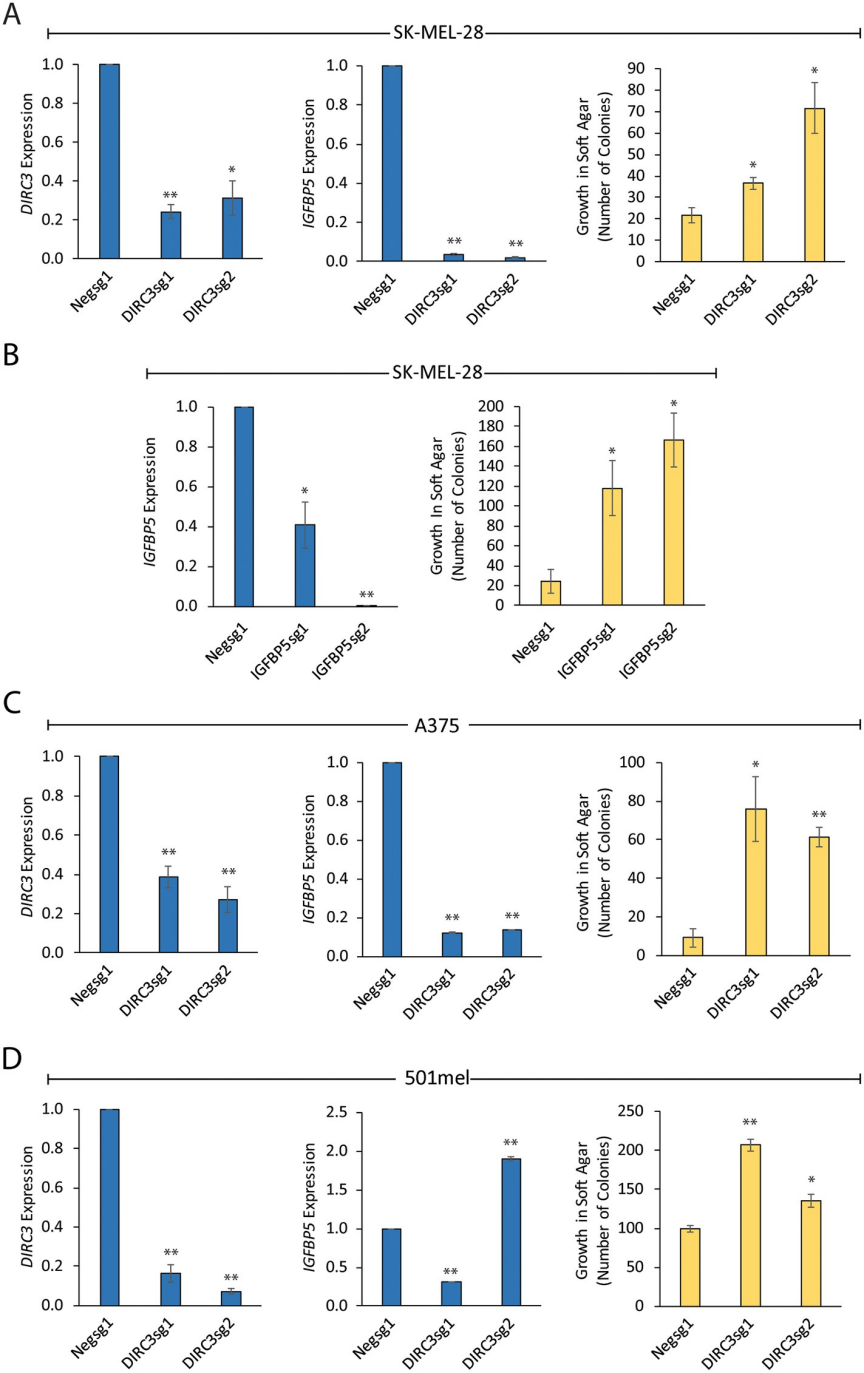
*DIRC3* acts through *IGFBP5* to block anchorage-independent growth in melanoma. Stable *DIRC3* (A) and *IGFBP5* (B) loss-of-function SK-MEL-28 cells were generated using CRISPRi and *DIRC3* and *IGFBP5* expression measured using RT-qPCR. Knockdown and control cell lines were seeded on soft agar and colony formation quantified 21 days later using ImageJ. Mean values +/- SEM, n = 3 (right panels). *DIRC3* depletion increases the anchorage-independent growth of multiple melanoma cell lines in soft agar. *DIRC3* expression was depleted in A375 (C) and 501mel (D) human melanoma cells using CRISPRi with two independent *DIRC3* targeting sgRNAs and a non-targeting negative control. *DIRC3* and *IGFBP5* levels were determined using RT-qPCR and expression changes shown relative to control (set at 1). Results are presented as mean values +/- SEM, n = 3. One-tailed student’s t-test p < 0.05 * p < 0.01 **. Knockdown and control cell lines were seeded on soft agar and colony formation quantified 21 days later using ImageJ. Mean values +/- SEM, n = 3 (right panels).

We next used CRISPRi to generate *DIRC3* loss-of-function cell lines and investigate the role of *DIRC3* in controlling cellular transformation in melanoma. To do this, *DIRC3* expression was first depleted in SK-MEL-28 cells by stable transfection of a plasmid co-expressing the dCas9-KRAB transcriptional repressor along with either the *DIRC3* targeting sgRNAs (*DIRC3*sg1 and *DIRC3*sg2) or a negative control. We generated two independent clonal lines in which *DIRC3* expression was depleted by (~70%) compared to a negative control (Fig 5A, left panel). These lines also displayed reduced *IGFBP5* (Fig 5A, middle panel) and proliferated to similar levels as control cells under normal growth conditions (S4 Fig). As anchorage-independent growth is a good predictor of melanoma metastasis *in vivo* [39], we assayed the effect of *DIRC3* loss-of-function on growth in soft agar. We found that stable reduction of *DIRC3* expression, using different dCas9-KRAB targeting sgRNAs, led to a significant increase in melanoma cell colony formation in soft agar (Fig 5A, right panel). We then investigated whether *IGFBP5* knockdown phenocopies the effect of *DIRC3* on anchorage-independent growth. We generated two stable *IGFBP5* knockdown SK-MEL-28 cell lines using CRISPRi with different sgRNAs in which *IGFBP5* levels were reduced by ~60% and ~99% (Fig 5B, left panel). The results revealed that *IGFBP5* loss-of-function induces a dose dependent increase in anchorage-independent growth in soft agar (Fig 5B, right panel). This is similar to the effect of *DIRC3* depletion and is consistent with our transcriptomic experiments showing that *DIRC3* activates *IGFBP5* to control shared gene expression programmes involved in cancer (Fig 4C and 4E). *DIRC3* therefore appears to act through *IGFBP5* to regulate growth in soft agar and exert its tumour suppressor effect.

We then assessed the generality of *DIRC3*-mediated control of anchorage-independent growth and extended our loss-of-function analysis to include additional *DIRC3* expressing melanoma cell lines. The results showed that CRISPRi mediated stable depletion of *DIRC3* in A375 and 501mel melanoma cells, using either *DIRC3*sg1 or *DIRC3*sg2 to target dCas9-KRAB to the *DIRC3* promoter, also resulted in significantly increased colony formation in soft agar compared to control (Fig 5C and 5D). This indicates that *DIRC3* regulation of anchorage-independent growth in melanoma is cell line-independent and is in agreement with TCGA clinical data showing that melanoma patients expressing low levels of *DIRC3* display decreased survival, compared to those classified based on high *DIRC3* expression (Fig 1D). Additionally, RT-qPCR analysis revealed that *IGFBP5* expression was suppressed in both A375 and one of the 501mel *DIRC3* loss-of-function cell lines. (Fig 5C and 5D, middle panels). Furthermore, the 501mel clonal cell line in which *IGFBP5* expression was no longer depleted showed the smallest increase in colony number (Fig 5D). Taken together, these results define *DIRC3* as a new melanoma tumour suppressor gene that acts through *IGFBP5* to inhibit the anchorage-independent growth of melanoma cells in culture.

### *DIRC3* regulates local chromatin structure to block SOX10 DNA binding and activate *IGFBP5*

**Fig 6:**
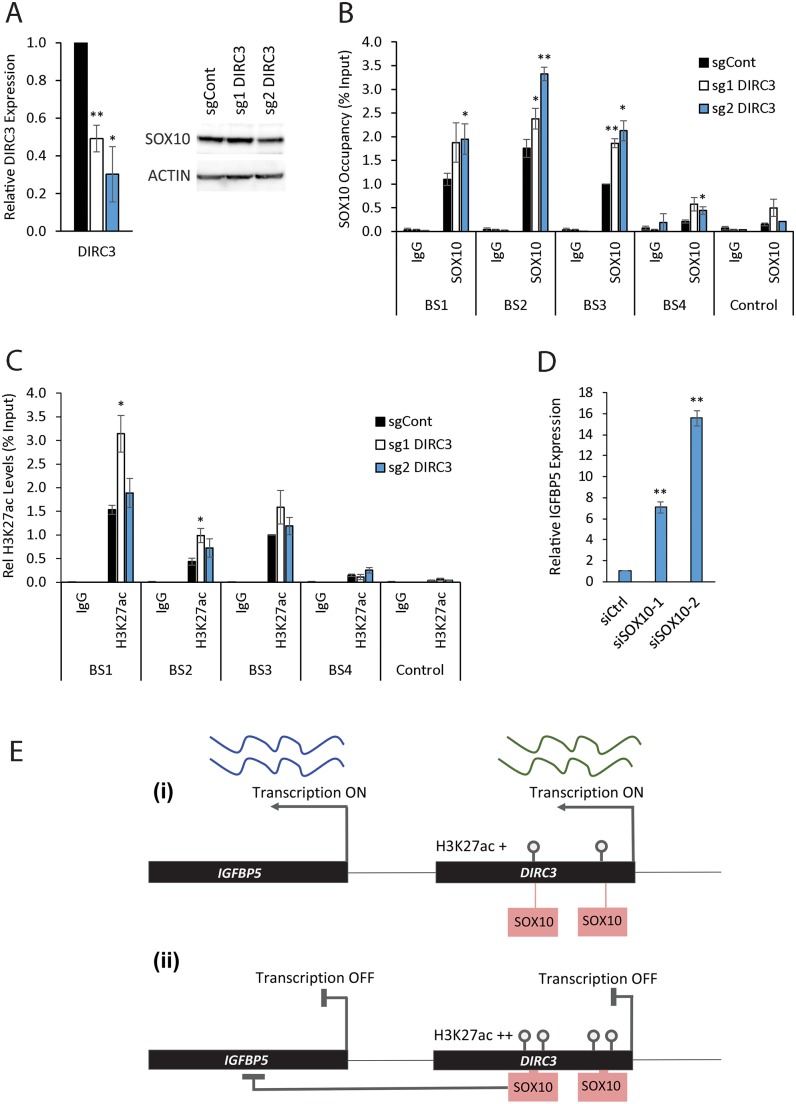
*DIRC3* induces closed chromatin at its site of expression thereby blocking SOX10 DNA binding and activating *IGFBP5*. ChIP assays were performed in *DIRC3* depleted SK-MEL-28 and control cell lines using the indicated antibodies against either SOX10, H3K27ac or an isotype specific IgG control. (A) *DIRC3* depletion was confirmed using qRT-PCR. Western blotting showed that SOX10 protein levels do not change upon *DIRC3* knockdown. ACTIN was used as a loading control. (B) The indicated SOX10 binding sites were analysed by qPCR. % input was calculated as 100*2^(Ct Input-Ct IP). (C) *DIRC3* depletion leads to an increase in H3K27ac levels at SOX10 bound regulatory elements within the *DIRC3* locus. (D) *SOX10* represses *IGFBP5* expression. *SOX10* was reduced in SK-MEL-28 cells using transfection of two independent siRNAs (see Fig 2C for SOX10 levels). *IGFBP5* expression was quantified using RT-qPCR three days later. *POLII* was used as a reference gene and expression changes are shown relative to a non-targeting control (set at 1). (E) Model illustrating that *DIRC3* acts locally to close chromatin and prevent SOX10 chromatin binding at melanoma regulatory elements within its locus. This leads to a block in SOX10 mediated repression of *IGFBP5* and subsequent increase in *IGFBP5* expression. All qPCR results are presented as mean values +/- SEM, n = 3. One-tailed student’s t-test p < 0.05 * p < 0.01 **.

MITF-SOX10 co-occupancy marks active regulatory elements within transcriptional enhancers in melanoma cells [29]. The *DIRC3* locus overlaps multiple MITF-SOX10 bound sequences and peaks of open chromatin structure (Fig 2A). *DIRC3* thus represents an exemplar to test whether MITF-SOX10 bound lncRNAs can modulate the downstream MITF-SOX10 transcriptional response in melanoma by regulating the association of these transcription factors with their binding sites in chromatin. To do this, we performed ChIP-qPCR in control and *DIRC3* depleted cells. We were unable to identify an anti-MITF antibody to work effectively in ChIP and so used SOX10 to determine changes in transcription factor occupancy at its binding sites within the *DIRC3* locus. We first confirmed SOX10 binding at the sites mapped by ChIP-seq within the *DIRC3* locus and at the *DIRC3* promoter in SK-MEL-28 cells. We then found that SOX10 chromatin occupancy was significantly increased at its binding sites following CRISPRi mediated stable depletion of *DIRC3* even though total SOX10 protein levels were not affected (Fig 6A and 6B). *DIRC3* therefore functions locally at its site of synthesis to block SOX10 binding at putative regulatory elements within its locus.

Active transcriptional regulatory elements are marked by H3K27ac modified chromatin. ChIP-qPCR showed that the SOX10-bound sites at *DIRC3* are specifically enriched for H3K27ac when compared to both an isotype control antibody and a non-bound region within the *DIRC3* locus (Fig 6C). These locations are thus likely to represent DNA sequences involved in transcriptional control in melanoma. Furthermore, stable depletion of *DIRC3* using CRISPRi led to an increase in the H3K27ac open chromatin mark at a subset of SOX10 bound sequences within *DIRC3* in SK-MEL-28 cells using ChIP-qPCR (Fig 6C). This suggests that *DIRC3* functions at its site of expression to close local chromatin structure and control transcription factor occupancy.

We then investigated whether *IGFBP5* is a *SOX10* transcriptional target and showed using RT-qPCR that *SOX10* depletion using two different siRNAs (see Fig 2C for *SOX10* levels) strongly increased *IGFBP5* expression approximately 7- and 16-fold in SK-MEL-28 cells (Fig 6D). These data suggest that SOX10 directly represses *IGFBP5* expression in melanoma. Although we can’t rule out the possibility that *DIRC3* also regulates *IGFBP5* independently of SOX10, our results are consistent with a model in which *DIRC3* acts locally to block SOX10 chromatin binding at melanoma regulatory elements and activate *IGFBP5* expression (Fig 6E). We propose that MITF-SOX10 bound lncRNAs, such as *DIRC3*, which function using this mechanism of action have potential to modify the MITF-SOX10 transcriptional response in melanoma.

## Discussion

MITF and SOX10 co-occupy approximately 3,600 binding sites on chromatin in human melanoma cells, marking a subset of active regulatory elements that function to control key melanoma transcriptional programmes underpinning proliferation, invasion and metastasis [29]. Previous studies have largely focussed on the ability of MITF-SOX10 to exert their effect on melanoma biology via regulation of protein-coding genes. Here we show that lncRNAs are also important components of MITF-SOX10-driven transcriptional programmes and identify 245 candidate melanoma-associated lncRNAs whose loci are co-bound by MITF and SOX10. These genes are marked by an increased ratio of H3K4me1:H3K4me3 modified chromatin, when compared to all melanocyte lineage lncRNAs identified in our study, suggesting that they preferentially overlap active enhancer-like transcriptional regulatory elements important for melanoma.

To exemplify the importance of MITF-SOX10-regulated lncRNAs in melanoma biology, we prioritised one, *DIRC3*. Significantly we found that *DIRC3* loss-of-function in three melanoma cell lines leads to increased anchorage-independent growth, a strong predictor of the metastatic potential of melanoma cells [39]. Mechanistically, we reveal that *DIRC3* is a nuclear regulatory lncRNA that functions in a transcript-dependent manner to activate expression of its adjacent gene, *IGFBP5*. We show that *IGFBP5* and *DIRC3* are located within the same self-interacting TAD in chromatin and demonstrate that *DIRC3* depletion leads to increased SOX10 occupancy and H3K27ac at putative regulatory elements within the *DIRC3* locus. Furthermore, *DIRC3* loss results in a concomitant increase in SOX10 mediated repression of *IGFBP5*. These data suggest that *DIRC3* acts at its site of synthesis to modify chromatin structure and control *IGFBP5* regulatory element activity in melanoma. This is important as dysregulated *IGFBP5* transcriptional control can act as a driver of cancer growth and metastasis, regulating cell proliferation, differentiation and metabolism using both *IGF1R*-dependent as well as -independent mechanisms of action [40, 41]. For example, regulatory elements within the 2q35 breast cancer susceptibility locus loop onto both *IGFBP5* and *DIRC3* whilst copy number alterations encompassing an *IGFBP5* enhancer on 2q35 modulate breast cancer risk [42, 43]. In melanoma, *IGFBP5* negatively regulates IGFR1 and MAPK kinase signalling to inhibit proliferation and metastasis [35] whilst a recent preprint reported that inactivation of *IGFBP5* distal enhancers down-regulates *IGFBP5* expression and promotes melanomagenesis by inducing an *IGF1R*-*AKT* signalling-dependent increase in glycolysis and metabolic reprogramming [44]. Given the key role of *IGFBP5* in melanoma biology, its regulation by *DIRC3* downstream from MITF-SOX10 is likely significant. Indeed, our results show that *IGFBP5* depletion phenocopies the increased anchorage-independent growth effect of *DIRC3* loss in SK-MEL-28 cells whilst others have reported that *IGFBP5* overexpression reduces anchorage-independent growth in A375 melanoma cells [35]. Moreover, we also discovered that *DIRC3* and *IGFBP5* common target genes are enriched for regulators of cancer associated processes such as cell migration, proliferation, metabolism and mesenchymal cell differentiation. *DIRC3* therefore appears to act through *IGFBP5* to exert its tumour suppressive effect in melanoma.

The ability of melanoma cells to switch between proliferative and invasive cell states is important for subpopulations of cells within a heterogeneous tumour to gain invasive properties and metastasize [31, 45]. High MITF-SOX10 expression defines the proliferative cell state in melanoma whilst invasive cells are MITF-SOX10 low. Our finding that *DIRC3* is transcriptionally repressed by MITF-SOX10 is therefore consistent with TCGA data showing that *DIRC3* levels correlate with an invasiveness gene expression signature in melanoma. This suggests that suppression of MITF-SOX10 during invasion of primary melanoma cells, the first step in metastatic colonization, up-regulates *DIRC3*. However, for metastases to form, melanomas must switch away from invasion towards proliferation. In the anchorage-independent growth assay, the initial invasive step is bypassed, and only the ability to grow in soft agar is assessed. In this assay, suppression of *DIRC3* expression increased anchorage-independent growth. Collectively these data are consistent with low MITF-SOX10 increasing *DIRC3* expression in invasive primary melanomas, but then at a site of metastatic colonization reestablishment of MITF-SOX10 would suppress *DIRC3* expression to allow proliferation to occur. This suggests that *DIRC3* may have dual roles at different stages of the disease and that down-regulation of *DIRC3* tumour suppressor function in metastatic melanomas may be an important event in melanoma progression. This is in line with the observation that TCGA melanoma patients with low *DIRC3* display decreased survival, since it is after all tumour expansion that drives patient death. Moreover, it has been shown that nutrient limitation or pseudo-starvation signalling mediated by inflammation can down-regulate MITF and drive invasion [46, 47]. In this context, in the presence of resource limitation, cells would need to proliferate slowly to balance reduced nutrient supply with demand, while becoming invasive to seek new nutrient supplies [48]. Once nutrient supply is restored, for example at a site of metastatic colonization, MITF expression would increase, and reduced *DIRC3* would enable proliferation to resume.

Phenotype switching between different proliferative and invasive states is accompanied by large scale changes in chromatin structure [45]. Our data suggests that MITF-SOX10 bound lncRNAs, as exemplified by *DIRC3*, may regulate chromatin accessibility at their sites of expression. Furthermore, the discovery that *DIRC3* blocks SOX10 binding to putative regulatory regions within its locus suggests that lncRNA components of MITF-SOX10 networks can act not only as downstream mediators of MITF-SOX10 function but as feedback regulators of MITF-SOX10 transcriptional activity. Such melanoma associated lncRNAs thus have potential to play widespread roles in fine tuning the MITF-SOX10 transcriptional response and may act as important regulators of cell state transitions in melanoma. In particular, our data indicates that *DIRC3* may be a novel clinically important melanoma tumour suppressor gene and we predict that driving up-regulation of *DIRC3* expression may represent a new therapeutic strategy for melanoma.

## Materials and methods

### Plasmid construction

To generate pX-dCas9-mod, dCas9 was excised from pAC94-pmax-dCas9VP160-2A-puro (Addgene #48226) as an *AgeI*-*FseI* fragment and subcloned into pX459 (Addgene #62988) to replace the nuclease active Cas9 protein. A modified sgRNA backbone was then synthesised as a gBlock (IDT) and introduced by cutting the vector with *BbsI* and *BamHI* and replacing the guide backbone with a *BsaI* and *BamHI* digested gene fragment that recapitulated the *BbsI* cloning sites. To generate pX-dCas9-mod-KRAB, the KRAB domain was PCR amplified using pHR-SFFV-dCas9-BFP-KRAB (Addgene #46911) as a template, and cloned into the *FseI* site in the pX-dCas9-mod vector. Five sgRNAs were designed against a genomic region −50 to +150 bp relative to the transcription start site of each target using the Zhang Lab CRISPR Design Tool (https://zlab.bio/guide-design-resources). Linker ligation was performed to insert each sgRNA as a *BbsI* fragment into either pX-dCas9-mod or pX-dCas9-mod-KRAB. We selected two effective sgRNAs for each target and used the published negative control sgRNA sequence described in [49]. To clone pCAGGS-*DIRC3*, full length *DIRC3* was synthesized as two gBlock Gene Fragments (IDT) and inserted into pGEM-T-Easy (Promega) using Gibson assembly. *DIRC3* was then PCR cloned as a *BglII* fragment into pCAGGS. Oligonucleotides used for cloning are shown in S7 Table.

### Cell culture and transfections

501mel, SK-MEL-28 and A375 human melanoma cells were cultured in 5% CO^2^ at 37°C in Dulbecco’s modified Eagle’s medium supplemented with 10% fetal bovine serum (growth medium). All cell lines were routinely tested for mycoplasma. For ASO LNA GapmeR (Exiqon) and siRNA (MITF, Sigma-Aldrich; SOX10, Dharmacon siGenome) mediated knockdown experiments, melanoma cells were seeded in a 6 well plate and transiently transfected using Lipofectamine 2000 reagent (Thermofischer) following the manufacturer’s instructions. 150 pmol each ASO or 100 pmol siRNA was transfected in each experiment. Experiments to analyse the effect of depletion were carried out 3 days after transfection. For CRIPSRi, 2μg plasmid DNA was transfected per well in a 6 well plate using Lipofectamine 2000 according to the manufacturer’s instructions. Three days later, cells were trypsinised, resuspended in growth medium containing 0.7 μg/ml puromycin and plated onto a 6-cm dish. Drug-resistant cells were grown for 7 days and harvested as a pool. To generate stable cell lines, individual drug resistant clones were isolated and expanded under selection using 1 μg/ml (SK-MEL-28), 1.3 μg/ml (501mel), or 1.5 μg/ml (A375). *DIRC3* expression in individual clones was characterised using RT-qPCR. To overexpress *DIRC3*, 250 ng pCAGGS-*DIRC3* or pCAGGS control were transfected into SK-MEL-28 cells using Lipofectamine 2000 and cells harvested for expression analysis 3 days later. siRNA and ASO sequences are listed in S7 Table.

### RT-qPCR

RNA was extracted using the GeneJET RNA purification Kit (ThermoFisher Scientific) and reverse transcribed using the QuantiTect Reverse Transcription Kit (Qiagen). Fast SYBR^™^ Green quantitative PCR was performed on a Step One^™^ Plus Real-Time PCR System (Applied Biosystems). The sequence of all RT-qPCR primers are shown in S7 Table.

### Western blotting and cellular fractionation

For Western Blot analysis, approximately 6x10^5^ cells were pelleted by centrifugation and lysed in 50μl of RIPA buffer (150 mM NaCl, 1% NP40, 0.5% EDTA, 0.1% SDS, 50 mM Tris pH 8, 0.5 mM protease inhibitor cocktail (Roche)) for 20 minutes at 4 °C with rotation. Samples were passed through a syringe needle, the supernatant was collected and protein concentration determined using a Bradford assay. 40 μg protein was loaded per well for SDS-PAGE using a Mini-PROTEAN Tetra cell system (BioRad) and Western blotting was performed with either anti-MITF antibody rabbit polyclonal (MERCK; HPA003259), anti-SOX-10 (Santa Cruz; sc-365692) or anti-β-ACTIN (Santa Cruz; sc-47778) mouse monoclonal antibodies. For cellular fractionation, approximately 1 x 10^6^ cells were harvested and pelleted at 4 °C. Cell pellet was re-suspended in 200 μl Lysis Buffer (15 mM HEPES, pH 7.5, 10 mM KCl, 5 mM MgCl2, 0.1 mM EDTA pH 8, 0.5 mM EGTA pH 8, 250 mM Sucrose, 0.4% Igepal, 1 mM DTT, 0.5 mM, protease inhibitor cocktail (Roche), 100 U/ml RNAsin) and incubated at 4 °C for 20 minutes with rotation to lyse. Cells were further disrupted by pipetting and centrifuged at 2000 g for 10 minutes at 4 °C. Nuclear (pellet) and cytoplasmic (supernatant) fractions were collected. RNA was then prepared from each fraction and analysed by RT-qPCR.

### Colony forming assays

Approximately 5x10^3^ *DIRC3/IGFBP5* depleted or control clonal cells were suspended in 1.5ml of growth medium containing 0.3% noble agar and plated on top of a 0.5% noble base agar layer in a six-well plate. Cells were then grown in 5% CO_2_ at 37°C and supplemented with 100 μl growth medium every three days. After 21 days cells were fixed in 1% formaldehyde and stained using 0.01% crystal violet. Colony number was quantified using ImageJ.

### Transcriptomics

For RNA-sequencing of Hermes melanocytes, IGR37 and IGR39 melanoma cells, cells were grown to 80% confluence and then harvested. Total RNA was purified using the RNeasy mini kit (QIAGEN) according to the manufacturer’s instructions including an on-column DNase digestion step. Library preparation and paired-end sequencing (44–59 million reads per sample) was performed at the Wellcome Trust Centre for Human Genetics, University of Oxford. LncRNA annotation from RNA-seq data was performed as described previously using CGAT pipelines [50, 51].

For gene expression analysis, total RNA was prepared in triplicate from knockdown (*DIRC3*, *IGFBP5*) and control SK-MEL-28 cells using the GeneJET RNA purification Kit (ThermoFisher Scientific). PolyA selected 150-bp paired end RNA sequencing was performed on the Illumina HiSeq4000 (Novogene). A minimum depth of 30M mapped reads were generated per sample. RNA-seq data was analysed as follows. Quality controls were applied for cleaning data for adapters and trimming of low quality sequence ends using trim_galore version 0.4.4. Cleaned data was aligned using FLASH v1.2.11 and mapped to the hg19 genome using bowtie version 1.1.2. The human gene annotation file was obtained from Gencode (v19, genome assembly hg19). Data were aligned to these annotations using the Bioconductor package GenomicAlignments version 1.34.0 function summarizeOverlaps. Statistical analysis was performed with the Bioconductor package DESeq2 (R version 3.5.0, DESeq version 1.22.2). Default settings were used. Differential gene expression was tested between knockdown and control groups. P-values were adjusted by the Benjamini-Hochberg method, controlling for false discovery rate (FDR). Gene Ontology analyses were performed using the Bioconductor package GOstats function hyperGtest. A list of all genes expressed in SK-MEL-28 cells was used as a background dataset. FDR cut-offs were computed from the resulting p-values using the brainwaver package function compute.FDR.

### Gene expression analysis of TCGA melanoma RNA-seq data

Expression correlations were performed as described in [52]. Expression data was retrieved using CGDS-R package (https://cran.r-project.org/web/packages/cgdsr/index.html). TCGA samples were ranked according to their expression value of the selected gene (x-axis gene) and plotted as relative expression in black line. The relative *DIRC3* expression (y-axis gene) for each sample was plotted as a bar graph in light grey and the moving average line with a window of 20 was plotted in the same colour as the y-axis. The Spearman correlation coefficient (rho) and an exact *P*-value of the correlation between the selected genes (x- and y-axis genes) were generated using R function ‘cor.test’.

### Chromatin immunoprecipitation

ChIP was performed as described in [9] using approximately 1x10^7^ SK-MEL-28 clonal CRISPRi cells per assay and 5 μg of the following antibodies: anti-SOX10 rabbit monoclonal antibody (Abcam; ab155279), anti-Histone H3K27ac rabbit polyclonal (Active Motif; #39133) or anti-rabbit IgG control (Merck; PP64). qPCR primers used to amplify SOX10 bound genomic sequences are shown in S7 Table.

### Chromatin analysis and datasets

H3K4me1, H3K4me3 and H3K27ac ChIP-seq chromatin modification data (GSE58953) from two tumorigenic melanoma cell lines were downloaded from [32]. ChIP-seq reads were processed and aligned to NCBI Build 37 (UCSC hg19) as described [32]. Reads mapping to lncRNA loci were further estimated using HTseq (version 0.9.1;—minaqual = 1, otherwise default parameters; [53]). HA-MITF and SOX10 ChIP-seq (GSE61967) data from [29] were used in this study. NHEK topologically associating domains (TADs) and loop annotated from HiC data were obtained from [36]. HiC heatmap was generated using JuiceBox [54]. Statistical analyses were performed using the R software environment for statistical computing and graphics (http://www.R-project.org/).

### Data Availability

RNA-seq data for *DIRC3 and IGFBP5* depleted SK-MEL-28 cells (GSE129467) and Hermes melanocytes, IGR37 and IGR39 melanoma cells (GSE129078) have been deposited in the NCBI GEO database (https://www.ncbi.nlm.nih.gov/geo/) under the indicated accession numbers. Numerical data underlying the individual figures are shown in S8 Table.

## Supporting information

S1 Fig MITF-SOX10 bound lncRNAs are enriched at enhancer-like regions. Distribution of the number of normalised H3K4me1 (left panel), H3K27ac (right panel) and H3K4me1:H3K4me3 ratio (middle panel) sequencing reads mapped to MITF-SOX10 bound lncRNAs (red) and all expressed lncRNA loci (grey) in an additional tumorigenic cell line (sh-PTEN PMEL cells). Differences between groups were tested using a two-tailed Mann-Whitney U test, and p-values are indicated.
(TIF)

S2 Fig Schematic illustration of the positionally equivalent mouse *DIRC3* and neighbouring protein coding genes (GRCm38/mm10). (TIF)

S3 Fig MITF and SOX10 repress DIRC3 in human melanoma cells. MITF and SOX10 were depleted in SK-MEL-28, 501mel and A375 cells using siRNA transfection. (A) MITF and (B) SOX10 protein levels were analysed by Western blotting. ACTIN was used as a loading control.
(TIF)

S4 Fig *DIRC3* depleted SK-MEL-28 cells proliferate to similar levels as control. *DIRC3* CRISPRi and control clonal knockdown SK-MEL-28 cells were seeded at a density of 1500 cells per well in a 6-well plate and grown at 37°C in 5% CO2. The number of cells were counted after 2 and 4 days. n = 3. Mean values +/- SEM.
(TIF)

S1 Table All melanocyte and/or melanoma expressed lncRNAs. (XLSX)

S2 Table MITF-SOX10 bound lncRNAs. (XLSX)

S3 Table *DIRC3* regulated genes (FDR 5%). (XLSX)

S4 Table *IGFBP5* regulated genes (FDR 5%). (XLSX)

S5 Table *DIRC3*-*IGFBP5* shared target genes. (XLSX)

S6 Table GO categories significantly enriched in shared *DIRC3*-*IGFBP5* targets (FDR 20%). (XLSX)

S7 Table Oligonucleotides. (XLSX)

S8 Table Numerical data used to generate individual figures. (XLSX)
