# Supplementary figures and images for: The MITF-SOX10 regulated long non-coding RNA DIRC3 is a melanoma tumour suppressor
Source: PLoS Genet. 2019 Dec 27;15(12):e1008501. doi: 10.1371/journal.pgen.1008501 (PMC6934268; doi:10.1371/journal.pgen.1008501)

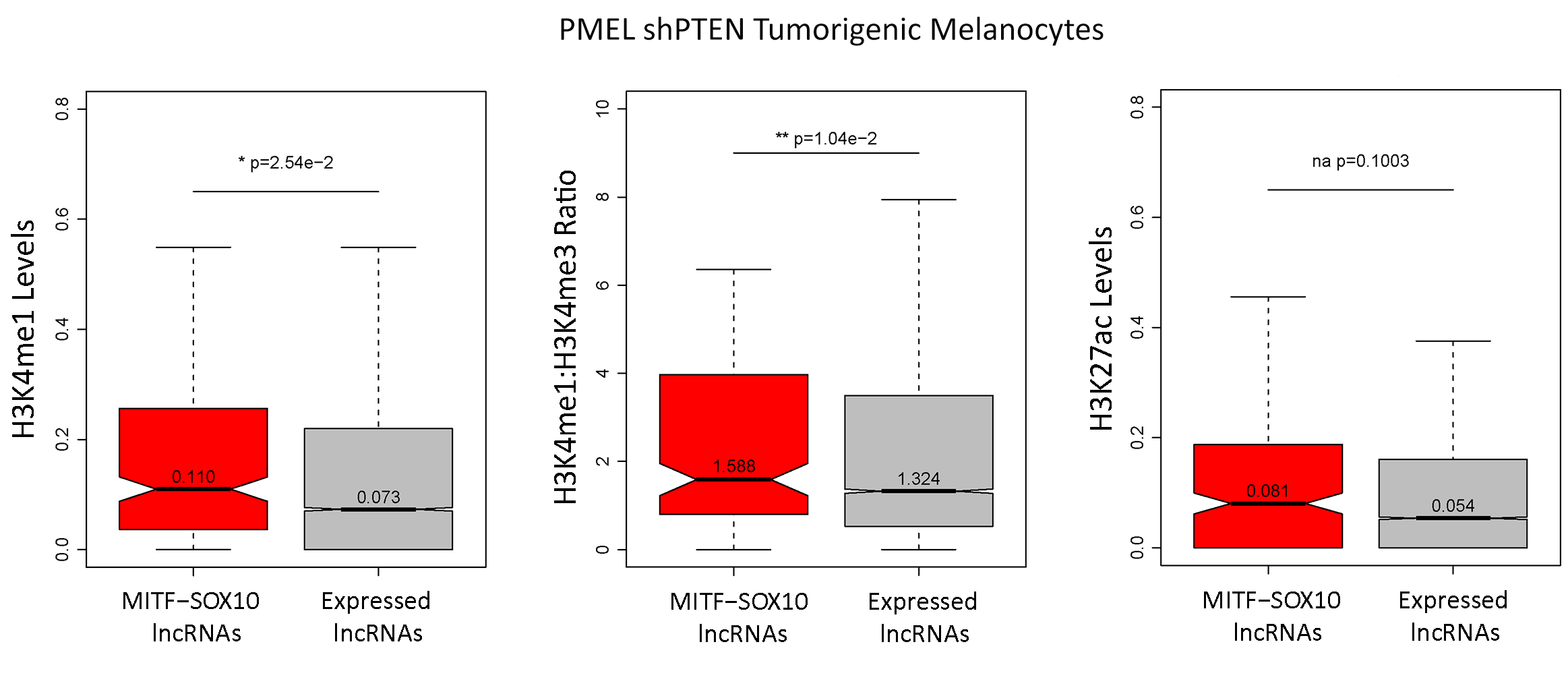

Supplement: S1 Fig — Distribution of the number of normalised H3K4me1 (left panel), H3K27ac (right panel) and H3K4me1:H3K4me3 ratio (middle panel) sequencing reads mapped to MITF-SOX10 bound lncRNAs (red) and all expressed lncRNA loci (grey) in an additional tumorigenic cell line (sh-PTEN PMEL cells). Differences between groups were tested using a two-tailed Mann-Whitney U test, and p-values are indicated. (TIF) [file pgen.1008501.s001.tif]

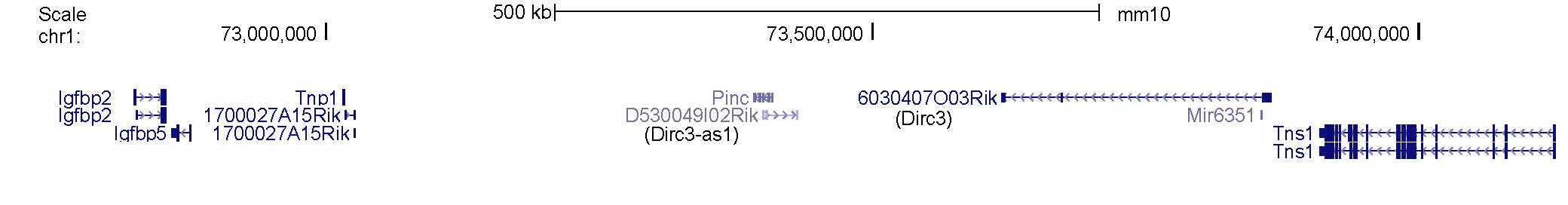

Supplement: S2 Fig — (TIF) [file pgen.1008501.s002.tif]

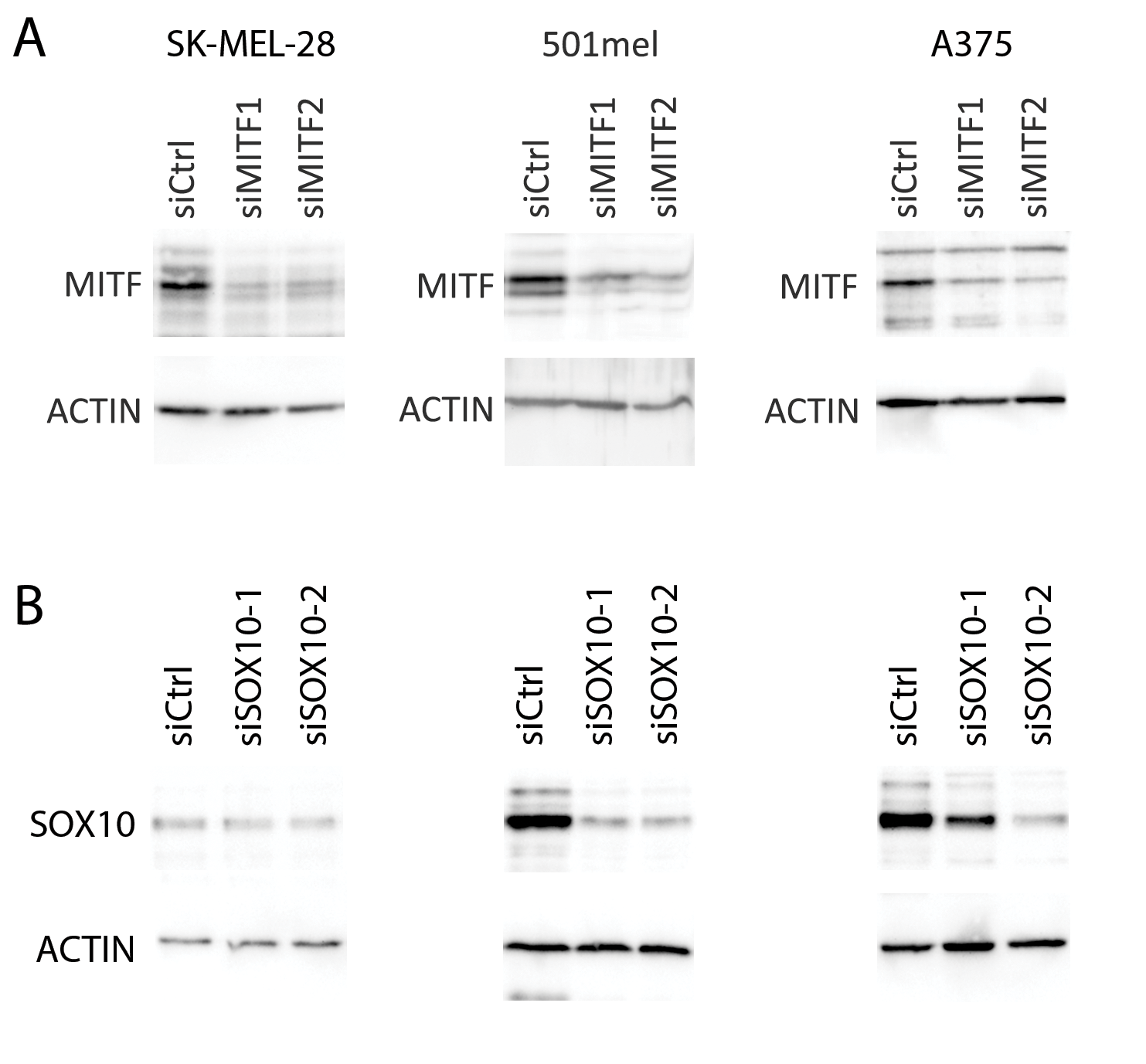

Supplement: S3 Fig — MITF and SOX10 were depleted in SK-MEL-28, 501mel and A375 cells using siRNA transfection. (A) MITF and (B) SOX10 protein levels were analysed by Western blotting. ACTIN was used as a loading control. (TIF) [file pgen.1008501.s003.tif]

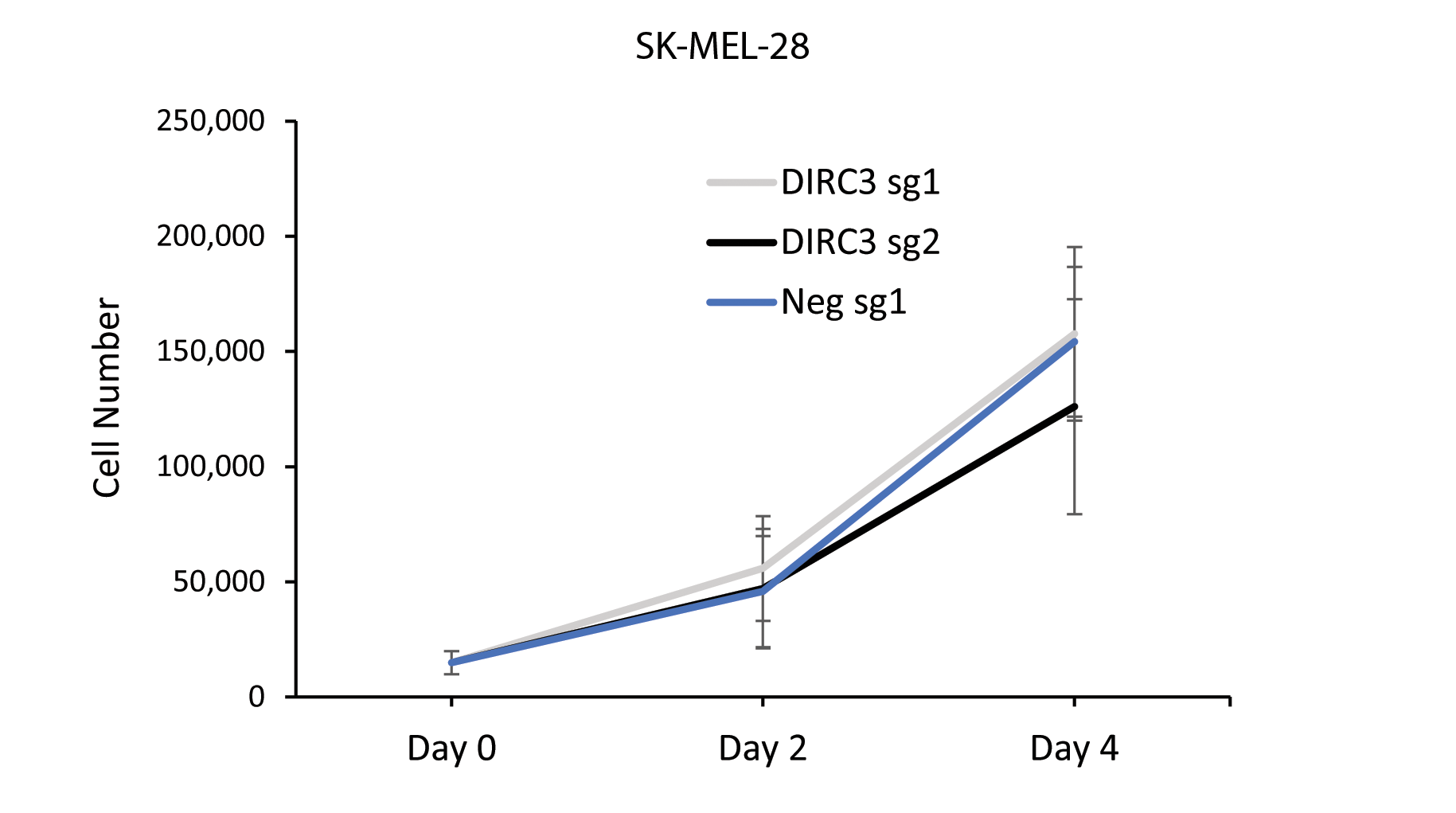

Supplement: S4 Fig — DIRC3 CRISPRi and control clonal knockdown SK-MEL-28 cells were seeded at a density of 1500 cells per well in a 6-well plate and grown at 37°C in 5% CO2. The number of cells were counted after 2 and 4 days. n = 3. Mean values +/- SEM. (TIF) [file pgen.1008501.s004.tif]
